# Editorial: Influence of Protein-Protein Interactions (PPIs) on the Outcome of Viral Infections

**DOI:** 10.3389/fmicb.2022.943379

**Published:** 2022-06-27

**Authors:** Rohit K. Jangra, Mercè Llabrés, Pablo Guardado-Calvo, Eva Mittler, Gorka Lasso

**Affiliations:** ^1^Department of Microbiology and Immunology, Louisiana State University Health Sciences Center-Shreveport, Shreveport, LA, United States; ^2^Department of Mathematics and Computer Science, University of the Balearic Islands, Palma, Spain; ^3^Structural Virology Unit, Institut Pasteur, Université de Paris Cité, CNRS UMR 3569, Paris, France; ^4^Department of Microbiology and Immunology, Albert Einstein College of Medicine, Bronx, NY, United States

**Keywords:** virus-host protein-protein interactions, PPI, amino acid variation, protein binding affinity, antibody recognition, virus-host PPI network, PPI database, viral infection

In the last two decades, the (re)emergence of zoonotic viruses [e.g., Severe acute respiratory syndrome coronavirus (SARS-CoV), Middle-Eastern respiratory syndrome coronavirus (MERS-CoV), SARS-CoV-2, H1N1 and H5N1 influenza viruses, Ebola virus, and Zika virus] has resulted in devastating consequences from a health, economic, and social perspective. Changes in ecological and environmental factors, demographics, and socio-economic behavior have increased the risk of spillover events and of (re)emergence of zoonotic viruses (Ahmed et al., 2019; Gibb et al., 2020; Johnson et al., 2020; Carlson et al., 2022). It is therefore imperative to establish preventive and therapeutic measures, as well as epidemiological surveillance to mitigate the effect of future outbreaks (Abubakar et al., 2012; Watsa, 2020).

Viruses are genetic parasites that exploit the host's molecular machinery by employing specific virus-host protein-protein interactions (PPIs) that mediate critical steps in virus replication and immune evasion. Thus, PPIs are prime targets for the development of therapeutics and vaccines. However, their characterization is an urgent albeit challenging task that benefits drastically from the integration of computational and experimental approaches. This Research Topic brings together nine articles (including original research and review articles) that collectively leverage experimentally and computationally derived information to describe important biological processes mediated by virus-host PPIs.

Opening this Research Topic, four review articles present an overview of virus-host PPIs and their biological role by focusing on specific host proteins, virus family or providing a more holistic view. Chung and Song summarize interactions of proteins expressed by oncogenic gammaherpesviruses [e.g., Epstein-Barr virus (EBV), Kaposi's sarcoma-associated herpesvirus (KSHV), and murine gammaherpesvirus 68 (MHV-68)] with host poly (ADP-Ribose) polymerase 1 (PARP1), a nuclear enzyme that regulates diverse cellular pathways. PARP1's interaction with several viral proteins supports establishment of viral latency by down-regulating viral DNA replication, and reducing virus production to prevent reactivation. Simultaneously, these viruses also employ multiple mechanisms to down-regulate PARP1 expression to then promote their own replication. Fishburn et al. systematically review virus-host PPIs that mediate virus entry and replication of various flaviviruses, including dengue virus (DENV), Zika virus (ZIKV), West Nile virus (WNV), yellow fever virus (YFV), and Japanese encephalitis virus (JEV). The authors also summarize the role of virus-host and intra-host PPIs mediated by cellular proteins involved in autophagy, mitochondrial, and innate immune responses including the antagonism of host immunity. Experimentally determined virus-host PPIs are compiled in PPI databases. Saha et al. give a detailed summary of virus-host PPI repositories and illustrate how publicly available data can be leveraged to identify shared and unique strategies employed by four emerging viruses to co-opt cellular processes. Notably, Saha et al. and Gabriel Valiente emphasize the poor overlap between the different repositories, highlighting the need to use meta-databases that combine different primary resources and inspect annotated PPIs during dataset assembly.

Experimental characterization of the virus-host protein interactome is far from complete and only few viruses have been extensively studied. In order to narrow this knowledge gap, computational tools can provide a catalog of high-confidence PPI predictions to be tested experimentally (Lasso et al., 2019). Recently, structural bioinformatics has experienced a major breakthrough by the introduction of Deep Learning (DL) methods to predict protein structure and PPIs from sequence (Yang et al., 2020; Baek et al., 2021; Jumper et al., 2021; Kryshtafovych et al., 2021; Bryant et al., 2022; Evans et al., 2022; Gao et al., 2022). Yang et al. summarize technical details of DL in the context of viral-host PPI prediction, including the different types of architecture, dataset preparation, feature engineering and performance assessment. While we expect DL-based methods to play a major role in inter-species PPI prediction in the near future, Yang et al. highlight important aspects of the technique that require further improvements and careful examination.

The following research articles illustrate important aspects of virus-host PPIs, including amino acid variations at protein interfaces, through a wide range of approaches such as X-ray crystallography, cryo-electron microscopy (Cryo-EM), molecular dynamics (MD), protein structure modeling, and binding affinity assays. Ford et al. combined DL-based protein structure modeling and protein docking to evaluate the potential binding between the spike (S) protein of the Omicron variant of SARS-CoV-2 and four neutralizing monoclonal antibodies (mAbs) targeting S with known structure. This study highlights amino acid variations that are predicted to decrease mAb-binding affinities without completely abrogating interactions and has important implications in the rapid assessment of neutralization escape potential of emerging viral strains. Zhu et al. experimentally studied the interaction between a PDZ-domain binding motif (PBM) found in SARS-CoV-2 envelope (E) protein and PDZ-containing cellular proteins, which are commonly targeted by other viruses (Javier and Rice, 2011). The authors identified an interaction of E protein with several PDZ domains of host proteins involved in cellular junctions and cell polarity, resulting in the sequestration of these host proteins in the Golgi compartment. Structural studies on PDZ:PBM complexes highlighted structure and sequence preferences at the interface. Ongoing studies focus on a point mutation in E protein localized in proximity to its PBM in the SARS-CoV-2 variant of concern beta that was shown to influence the binding affinity of E protein for PDZ domains. Glycans also play a key role in modulating the interaction with host proteins (Thompson et al., 2019; Watanabe et al., 2019). However, their intrinsic flexibility and cell-type specific composition makes them difficult to study experimentally. Stagnoli et al. combine Cryo-EM and MD to investigate the composition and dynamics of the glycan shield in the SARS-CoV-2 S protein. The authors show that the conformation of the glycans that best fit the Cryo-EM density map are those in which the movement of the most external carbohydrates are more geometrically restricted, providing an understanding of why these glycans are visible by Cryo-EM. Finally, Sabariegos et al. describe how the interaction between the cellular kinase Akt and the Hepatitis C virus (HCV) RNA-dependent RNA polymerase NS5B modulates this viral protein via phosphorylation of conserved residues. Site-directed mutagenesis of key NS5B residues to mimic phosphorylation significantly reduced RNA polymerase activity and prevented rescue of HCV from infectious clones, thus, describing a mechanism of viral polymerase inactivation whose biological role remains to be determined.

In conclusion, this Research Topic provides an overview of computational and experimental approaches that, when combined, can significantly accelerate our understanding of virus-host PPIs and their biological role in viral infectious diseases. We are grateful for the valuable contributions of authors, reviewers, and members of the Editorial team at Frontiers.

## Author Contributions

All authors listed have made a substantial, direct, and intellectual contribution to the work and approved it for publication.

## Funding

RKJ would like to acknowledge funding from Louisiana State University Health Sciences Center-Shreveport and National Institute of Allergy and Infectious Diseases of the National Institutes of Health (R21 AI156482).

## Conflict of Interest

The authors declare that the research was conducted in the absence of any commercial or financial relationships that could be construed as a potential conflict of interest.

## Publisher's Note

All claims expressed in this article are solely those of the authors and do not necessarily represent those of their affiliated organizations, or those of the publisher, the editors and the reviewers. Any product that may be evaluated in this article, or claim that may be made by its manufacturer, is not guaranteed or endorsed by the publisher.
